# Telehealth use in patients with type 2 diabetes in Australian general practice during the COVID-19 pandemic: a retrospective cohort study

**DOI:** 10.3399/BJGPO.2021.0200

**Published:** 2022-06-01

**Authors:** Chisato Imai, Judith Thomas, Rae-Anne Hardie, Christopher Pearce, Tony Badrick, Andrew Georgiou

**Affiliations:** 1 Centre for Health Systems and Safety Research, Australian Institute of Health Innovation, Macquarie University, North Ryde, New South Wales, Australia; 2 Outcome Health, East Burwood, Victoria, Australia; 3 Royal College of Pathologist of Australasia Quality Assurance Programs, St Leonards, New South Wales, Australia

**Keywords:** general practice, COVID-19, diabetes mellitus, type 2, telemedicine

## Abstract

**Background:**

The Australian government introduced temporary government-subsidised telehealth service items (phone and video-conference) in mid-March 2020 in response to the COVID-19 pandemic. The uptake of telehealth by patients with type 2 diabetes (T2DM) for consulting with GPs is unknown.

**Aim:**

To evaluate the uptake of telehealth consultations and associated patient characteristics in Australian general practice, including the frequency of haemoglobin A1c (HbA1c) tests and change in HbA1c levels by telehealth use, compared with guideline recommendations.

**Design & setting:**

This exploratory study used electronic patient data from approximately 800 general practices in Victoria and New South Wales (NSW), Australia. A pre-COVID-19 period from March 2019–February 2020 was compared with a pandemic period from March 2020–February 2021. Patients diagnosed with T2DM before March 2018 were included.

**Method:**

Telehealth uptake patterns were examined overall and by patient characteristics. Generalised estimating equation models were used to examine patient probability of 6-monthly HbA1c testing and change in HbA1c levels, comparing between patients who did and patients who did not use telehealth.

**Results:**

Of 57 916 patients, 80.8% had telehealth consultations during the pandemic period. Telehealth consultations were positively associated with patients with T2DM who were older, female, had chronic kidney disease (CKD), prescribed antidiabetic medications, and living in remote areas. No significant difference was found in 6-monthly HbA1c testing and HbA1c levels between telehealth users and patients who had face-to-face consultations only.

**Conclusion:**

Telehealth GP consultations were well utilised by patients with T2DM. Diabetes monitoring care via telehealth is as effective as face-to-face consultations.

## How this fits in

Understanding the use of telehealth consultation modalities in general practice for the ongoing management of diabetes during the COVID-19 pandemic represents a significant gap in current literature. This study investigates the use of telehealth consultations (predominantly via phone) in patients with T2DM and the patient characteristics, as well as the potential effects on the continuity of diabetes care, using a large cohort from approximately 800 Australian general practices. The findings based on HbA1c testing suggest that diabetes monitoring care via telehealth is as effective as face-to-face consultations.

## Introduction

As healthcare systems around the world responded to challenges arising from the COVID-19 pandemic, including acute care needs of patients presenting with COVID-19, there was increasing concern about maintaining existing healthcare services for the ongoing management of chronic diseases. Rapidly increasing infection rates necessitated the introduction of COVID-19 containment measures, including social distancing and lockdowns, to control transmission of the virus. However, such measures also had the potential to impact on the lifestyle, health, or ongoing management of patients with existing chronic conditions,^1^ including diabetes mellitus.^2^

Patients diagnosed with T2DM need to regularly monitor their diet, exercise, and blood glucose levels in partnership with their healthcare providers.^3^ In Australian primary care, GPs perform a fundamental role in the diagnosis and management of patients with diabetes,^4^ which includes HbA1c testing at least 6 monthly to monitor glycaemic control in patients with T2DM.^4^

During periods of lockdown, many countries facilitated continuity of care for patients with diabetes through rapid implementation or expansion of telehealth modalities such as telephone^5–11^ or video-consultations.^6–8^ Australia funds general practice through a universal health insurance scheme (Medicare), which subsidises fee-for-service activity.^12^ In the early stages of the pandemic, the Australian government implemented a staged rollout to greatly expand Medicare-subsidised telehealth services for GPs to conduct telephone or video telehealth consultations with their existing patients. While the rapid uptake of these new telehealth services has been reported for Australian general practice activity,^13^ it is not known to what extent these telehealth consultations were utilised by patients with T2DM, or whether this mode of consultation impacted diabetes care. Comparisons between the use of telehealth and face-to-face consultations for glycaemic control in patients with diabetes during the pandemic has been studied in outpatient settings in Japan,^10^ the US,^7^ South Korea,^9^ and Australia.^8^ However, these studies were based on limited populations (≤2 tertiary facilities or diabetes clinics) and short study periods (<6 months), and the results of the effects of telehealth on diabetes care were mixed.

Understanding of the use of telehealth consultation modalities in general practice for the ongoing management of diabetes, along with patient outcomes, represents an important aspect of continuity of care if telehealth is to remain part of general practice care. Hence, this study aimed to evaluate the use of telehealth consultations and the potential impact on the continuity of diabetes care, by assessing the following: (1) the uptake of telehealth consultations and associated patient characteristics; and (2) testing frequency compared with guideline-recommended 6-monthly HbA1c testing and HbA1c levels by consultation mode (telehealth versus face to face). This was an exploratory study utilising a large cohort from approximately 800 general practices to examine the telehealth use in patients with diabetes since telehealth became widely accessible to Australians during the COVID-19 pandemic.

## Method

### Study design and setting

The study period covered 2 years from March 2019–February 2021. Medicare-subsidised GP consultations via phone and video-conference were introduced in mid-March 2020, shortly after the COVID-19 pandemic was declared. The study period was separated into the following two intervals: the first year (March 2019–February 2020) was defined as the pre-COVID-19 period; and the second year (March 2020–February 2021) as the COVID-19 pandemic period. In this study, telehealth included phone and video-conference. Since the use of video-conference was limited (<0.1% of total consultations) in the study population, telehealth indicated here represents phone consultations.

### Participants

Inclusion criteria to be met by all study participants were as follows: (i) diagnosis with T2DM before March 2018 (to have had at least a 1-year history); (ii) active status (defined by the Royal Australian College of General Practitioners [RACGP]^14^ as individuals who had attended a practice three or more times in the past 2 years at the time of visit); and (iii) having at least one HbA1c test during the pre-COVID-19 period. As patients in Australia can visit more than one general practice, and may have died or moved during the study period, the latter two inclusion criteria were required to ensure that study patients attended a practice within the study catchment area for diabetes care.

### Data sources and definition

This study used non-identifiable electronic health records collected from approximately 800 general practices in Victoria and NSW, Australia (extracted in August 2021). Outcome Health, as the data custodians, routinely gather electronic data from general practices into the Population Level Analysis and Reporting (POLAR) Aurora research platform in a de-identified and secured format. POLAR data include patient demographics, Medicare service item numbers, diagnosis, pathology testing, and prescription medications. Details of the POLAR data are comprehensively documented elsewhere.^15^

Patients with T2DM were identified using Systematized Nomenclature of Medicine Clinical Terms (SNOMED CT) codes that fell into the concept of ‘diabetes mellitus type 2 (disorder)’ to identify T2DM.^16^ Study patients were also identified with CKD as one of the serious diabetes complications.^17^ The identification of CKD was based on SNOMED codes classified into ‘chronic kidney disease (disorder)'^16^ or pathology results (having ≥2 estimated glomerular filtration rate values <60 ml/min/1.73 m^2^ and/or ≥2 albumin-to-creatinine ratio values ≥3.5 mg/mmol for females, or ≥2.5 mg/mmol for males, at least 90 days apart).^18^

Drug therapies were identified based on the Anatomical Therapeutic Chemical classification codes^19^ in the prescription data. Prescriptions under the group of A10 (drugs used in diabetes) were used and categorised into the following three groups: insulin (A10-A); oral glucose-lowering agents only (A10-B); and no medication (that is, no A10 prescription records). An insulin group can include patients using the combined therapy with oral glucose-lowering agents.

GP consultation type (face-to-face and telehealth) was identified from item numbers in the Medicare service data.^20,21^ In this study, patients who had one or more consultations billed as telehealth GP consultations were considered telehealth users in comparison with patients who had GP consultations via face to face only (for example, a patient was classified a telehealth user if both telehealth and face-to-face consultations were used).

Australian guidelines consider HbA1c≤53 mmol/mol (7%) with a range of 48–58  mmol/mol as the target glucose level, with the recommendation of HbA1c testing every 6 months in patients with adequate glycaemic control and 3 months in patients with inadequate control.^4^ Thus, HbA1c levels and testing performance were evaluated in patients, comparing with the recommended threshold (HbA1c ≤53 mmol/mol) and frequency (at least once every 6 months allowing 15 days leeway).

### Statistical analysis

For the analysis of aim 1 (telehealth use and patient characteristics), the authors looked at the overall proportion (%) of telehealth consultations and the characteristics of patients who were telehealth users during the COVID-19 pandemic period. Patient characteristics included patient age, sex, socioeconomic status (SES), CKD, prescriptions for antidiabetic medications, residence remoteness (that is, city or regional or remote), and state. To evaluate the association between patient sociodemographic factors and the use of telehealth consultations, adjusted relative risks (RR) were estimated by using a generalised estimating equation (GEE) model with the Poisson distribution and the Huber–White Sandwich estimator.^22,23^ The GEE model included the covariates of patient factors (that is, age, sex, SES, remoteness, prescription, state, and CKD), with practice attended as a cluster and the exchangeable correlation structure.

For aim 2 (the patient probability (%) of ≤6-monthly HbA1c testing and mean HbA1c level by telehealth use during the pandemic period), the analysis used GEE models with the Poisson and Gaussian distributions, respectively. The outcome variable of each model was the binary outcome of ≤6-monthly testing (that is, yes and no) and the continuous variable of the mean HbA1c level for each patient. Both GEE models included practice attended as a cluster and the exchangeable correlation structure, with the covariates of telehealth use (that is, yes and no), patient characteristics, the total number of GP consultations, and mean HbA1c value measured during the pre-COVID-19 period. For subgroup analyses, patients were examined by the adequacy of glycaemic control (≤53 mmol/mol) before the pre-COVID-19 period. All analyses were performed in R (version 4.0.2).

## Results

### Study patients

A total of 113 569 patients with T2DM were identified who had visited a general practice at one point in time from March 2019–February 2021, of which 70 675 patients attended actively throughout the 2 years. After excluding 12 759 patients with no records of HbA1c tests during the pre-COVID-19 period, 57 916 patients were finally selected as the study cohort. As a sensitivity analysis, characteristics of patient demographics were compared between all identified patients with T2DM (*n* = 113 569) and the selected cohort (*n* = 57 916). Overall characteristics were similar between the two groups (Supplementary Figure S1).

### Telehealth use and patient characteristics

Of the total of 57 916 patients, the number of patients who claimed telehealth GP consultations was 114 (0.2%) and 46 783 (80.8%) during the pre-COVID-19 and pandemic periods, respectively.

**Figure 1.**
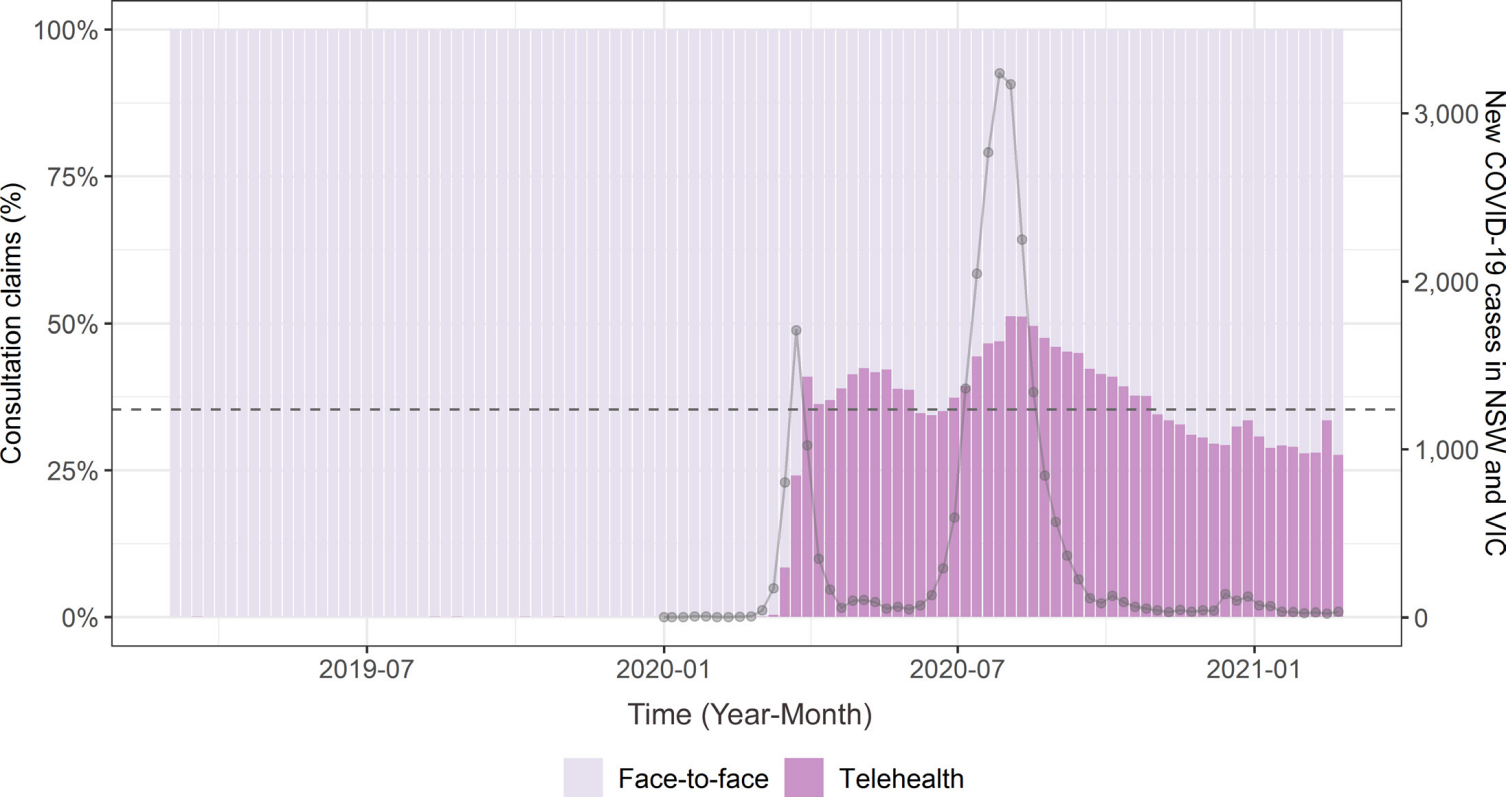
Telehealth uptake from March 2019–March 2021. Pink bars represent consultation types by percentage (left y-axis). The dashed horizontal line represents the mean of weekly total telehealth consultation claims during the COVID-19 pandemic period. The solid line with dots is the total reported number of new weekly COVID-19 cases for New South Wales (NSW) and Victoria (VIC) combined (right y-axis)

While the mean of telehealth consultation claims was only 0.03% of the weekly total claims (four out of 11 384) before the pandemic period, the proportion of telehealth consultations during the pandemic period was 35.7% (4499 out of total 12 595). The proportion of telehealth consultation claims peaked in August 2020 (Figure 1), when the number of COVID-19 positive cases increased in Australia. The mean HbA1c level in study patients during the pre-pandemic and pandemic periods were 54.5 mmol/mol (95% confidence interval [CI] = 54.4 to 54.6) and 55.6 mmol/mol (95% CI = 55.4 to 55.7), respectively.

Table 1 provides the demographic characteristics of study participants who had telehealth consultation claims during the pandemic period. Telehealth consultations were more likely to be used by patients with diabetes if they were older (for example, aged 65–74 years versus <65: RR 1.02, 95% CI = 1.01 to 1.03), female (RR 1.06, 95% CI = 1.05 to 1.07), residing in regional or remote areas (RR 1.05, 95% CI = 1.03 to 1.07) or Victoria (RR 1.19, 95% CI = 1.14 to 1.23), had CKD (RR 1.04, 95% CI = 1.03 to 1.05), or prescribed antidiabetic medications (for example, none versus oral-agents only: RR 1.05, 95% CI = 1.04 to 1.06). Strong evidence was not observed for the association between telehealth use and patient SES (for example, low versus high SES: RR 1.01, 95% CI = 1.00 to 1.03).

**Table 1.** The characteristics of patients who had a teleconsultation during the COVID-19 pandemic period (April 2020–March 2021)

		Number of patients			RR		
		Total	Telehealth	%	Estimate	95% CI	
**Total**		57 916	46 783	80.8			
**Age, years**	<65	17 979	13 892	77.3	Ref		
	65–74	18 405	14 801	80.4	1.02	1.01	1.03
	≥75	21 532	18 090	84.0	1.05	1.04	1.07
**Sex**	Male	31 569	24 844	78.7	Ref		
	Female	26 347	21 939	83.3	1.06	1.05	1.07
**SES**	Low	18 925	14 726	77.8	Ref		
	Middle	18 581	15 364	82.7	1.02	1.00	1.03
	High	20 410	16 693	81.8	1.01	1.00	1.03
**CKD**	None	45 088	35 966	79.8	Ref		
	Yes	12 828	10 817	84.3	1.04	1.03	1.05
**Prescription**	None	13 230	10 485	79.3	Ref		
	Oral-agents only	38 861	31 277	80.5	1.05	1.04	1.06
	Insulins	5825	5021	86.2	1.09	1.08	1.11
**Major city**	Yes	48 388	38 512	79.6	Ref		
	No	9528	8271	86.8	1.05	1.03	1.07
**State**	New South Wales	20 976	14 700	70.1	Ref		
	Victoria	36 940	32 083	86.9	1.19	1.14	1.23

CKD = chronic kidney disease. RR = relative risks. SES = socioeconomic status.

### HbA1c testing frequency and HbA1c level

**Figure 2.**
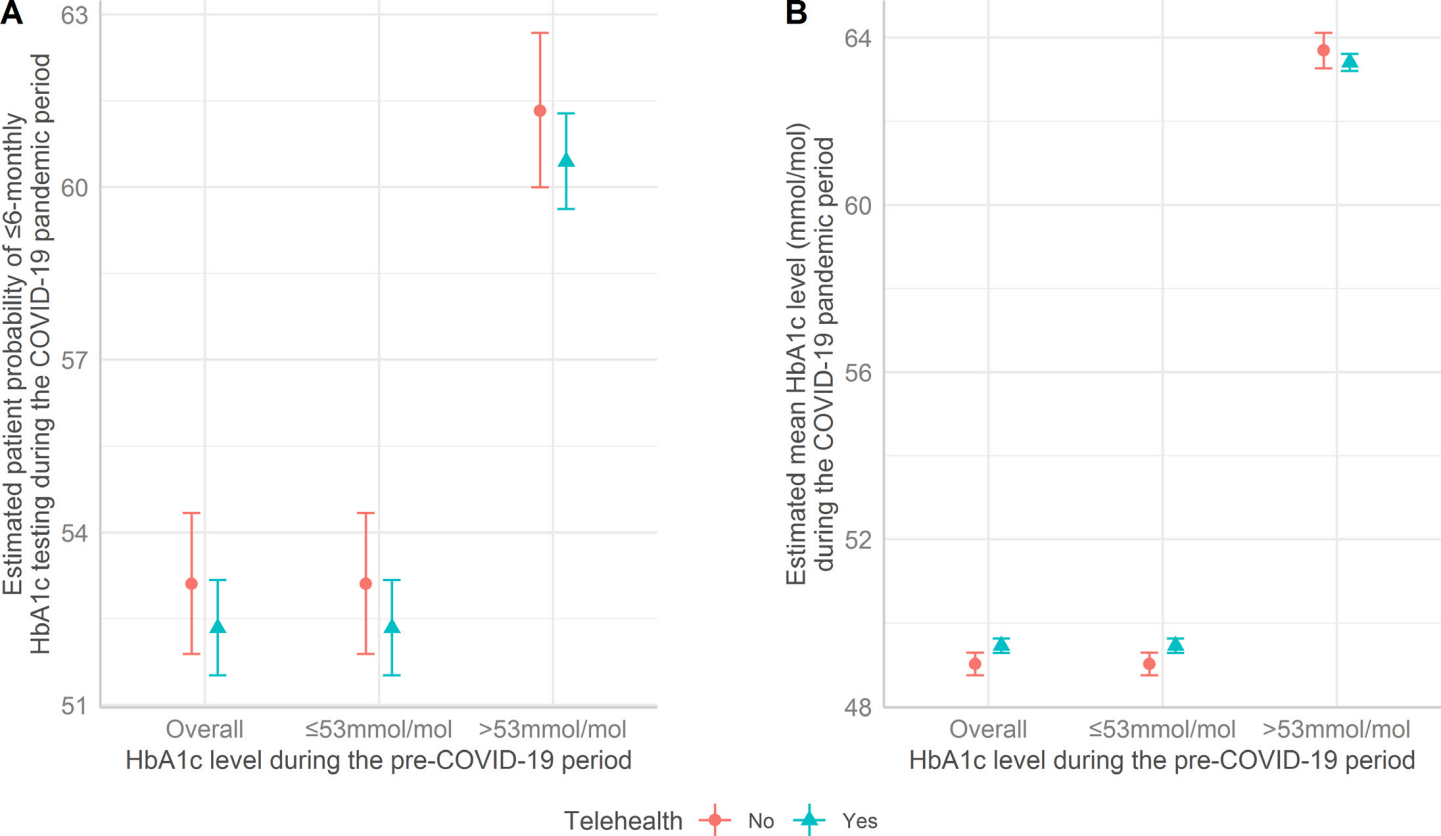
(**A**) Estimated testing probability for patients to carry out ≤6-monthly HbA1c tests and (**B**) estimated HbA1c level during the COVID-19 pandemic period

The estimated probability for patients to have carried out ≤6-monthly HbA1c tests during the pandemic period is presented in Figure 2A. Overall, patients who had suboptimal glycaemic control (that is, >53 mmol/mol) before the pandemic were more likely to conduct ≤6-monthly testing than patients with adequate glycaemic control (that is, ≤53 mmol/mol). The association between telehealth use and ≤6-monthly testing was not identified. For instance, in patients with adequate glycaemic control (that is, ≤53 mmol/mol) before the pandemic, the ≤6-monthly testing probability was 52.3% (95% CI = 51.5% to 53.2%) for individuals who did use telehealth consultations during the pandemic period and 53.1% (95% CI = 51.9%54.3%) for those who did not use telehealth.

Figure 2B provides the estimated mean of HbA1c levels during the COVID-19 pandemic period. Associations were not observed between telehealth use and HbA1c levels. In the subgroup of patients who had inadequate glycaemic control before the pandemic period, for instance, individuals who used telehealth had 63.4 mmo/mol (95% CI = 63.2 to 63.6 mmol/mol), whereas patients who did not use telehealth had 63.7 mmol/mol (95% CI = 63.3 to 64.1 mmol/mol) during the pandemic period. In patients who had adequate glycaemic control before the pandemic, the mean HbA1c level also did not differ by telehealth use (telehealth users 49.5 mmo/mol [95% CI = 49.3 to 49.6 mmol/mol]) versus no telehealth use (49.0 mmol/mol [95% CI = 48.7 to 49.3 mmol/mol]).

## Discussion

### Summary

The rapid adoption of telehealth GP consultations in patients with T2DM was observed after temporary Medicare-subsidised telehealth services were introduced in mid-March 2020. Overall, the majority of the patients with T2DM in the study (80.8%) used telehealth during the pandemic period, with higher uptake by individuals who were older, female, had CKD, or resided in remote or regional areas. The findings illustrate that telehealth was more likely to be utilised not only by patients who had geographically limited access to health care (remote or regional areas), but also those who were at a higher risk of serious COVID-19 complications (that is, older adults) and those who required close monitoring of care (that is, having a complication or antidiabetic prescription). Both the probability of carrying out ≤6-monthly HbA1c testing and HbA1c values did not change by consultation modality.

### Strengths and limitations

As the identification of factors was not the main objective of this study, one limitation in the study was that all possible factors for the uptake of telehealth consultations and HbA1c tests were not included such as other common diabetes complications (for example, cardiovascular disease, retinopathy), mental health conditions, or COVID-19 infections. The comparison between different telehealth modes (phone versus video) would have been also important to explore. While the analysis could not be pursued for the limited use of video-conference in this cohort, a systematic review study comparing the effects between video and phone consultations^24^ found that video had advantages in provider-related outcomes (for example, diagnosis accuracy, fewer readmissions) over phone consultations, which potentially suggests varying effects on patient outcomes by telehealth mode. Additionally, the study sites (NSW and Victoria) were most significantly impacted by COVID-19 in Australia. Thus, patients in the study states were more likely to have promoted telehealth use and the telehealth use in other states requires further studies. Another limitation is unavailability of other important diabetes care information such as electronic prescribing and specialist consultations. In Australia, electronic prescribing service became available with staged rollouts in 2020;^25^ however, the use of electronic prescribing in the study cohort could not be identified. Specialist data were also not available in this study as specialist care is generally provided outside general practice in Australia. Future studies on the utilisation of electronic prescriptions and telehealth for specialist care will be critical to elucidate the overall flow of monitoring care and treatment, as well as to understand the effectiveness and efficiency of remotely provided services within diabetes care.

Despite these limitations, there are substantial strengths in the study over existing research, including the large study population and comprehensive data, which include both patient demographic and longitudinal clinical information. A prior study using Australian general practice data from the POLAR platform has demonstrated the value of the data source for evaluating the quality of care and patient outcomes in a sample population approximating the Australian diabetes population.^26^ Furthermore, telehealth in Australia was primarily limited to certain patient populations (for example, between specialists and patients in remote areas, at home, or supported by rural clinics^27^) before the COVID-19 pandemic. The study is one of the first to utilise a large cohort and explore the telehealth use and potential effects on diabetes care since telehealth became widely available to all Australian patients. Thus, the study data are greatly beneficial in illustrating the general characteristics of diabetes care activities and the use of telehealth during the COVID-19 pandemic.

### Comparison with existing literature

Previously, factors such as older age, lower SES, and remote locations were considered potential barriers to delivering health care via telehealth owing to financial hurdles to internet use as well as limited infrastructure, internet skills, and acceptance of technology.^28^ However, the present study identified higher telehealth uptake in older patients as well as those of rural residence, in addition to no disparity of telehealth use by patient SES. The discrepancy of the findings may be partially explained by different study settings between the present study and the majority of existing literature. For instance, the predominant mode of telehealth in Australia is via telephone,^29^ which entails fewer technological challenges than video-conference. Furthermore, in the present study setting, telehealth GP consultations were covered by Medicare for Australian residents and utilised by a majority of patients with T2DM. On the other hand, telehealth was less commonly used before the COVID-19 pandemic, when prior studies were published. In the light of the COVID-19 pandemic, telehealth was rapidly adopted in the Australian healthcare system to address pandemic-associated challenges. As a result, the characteristics of patients utilising telehealth might have shifted in such a way that the present study observed patterns of higher telehealth uptake in the populations who were at higher risk of serious COVID-19 complications (that is, older patients) and requiring regular GP consultations (for antidiabetic prescriptions, or for CKD). A recent study conducted during the pandemic period in the US has similarly reported higher uptake of telehealth (phone) consultations in patients with diabetes of older age and using insulin.^7^

Given the recent emergence of the COVID-19 pandemic, there are few comparable studies using large population cohorts to comprehensively evaluate the potential effects of telehealth use in general practice on diabetes care during the pandemic. However, several studies based on cohorts from a tertiary or specialist care facility have investigated the effects of telehealth on glycaemic control. Although these studies reported mixed results, most studies reported either improvement^8,10,11^ or no significant changes^7^ in HbA1c levels associated with telehealth use during the pandemic. Systematic reviews^30,31^ published before the COVID-19 pandemic also reported the positive effects of telehealth on T2DM management, including a greater reduction in mean HbA1c levels compared with conventional face-to-face consultations. Overall, existing literature points to the potentially positive impact of telehealth for diabetes care, which corresponds to the findings outlined in this study.

### Implications for research and practice

The findings based on HbA1c testing suggest that diabetes care monitoring via telehealth is as effective as face-to-face consultations. Considering the effectiveness of telehealth on diabetes care, it may be also beneficial to promote more utilisations of telehealth to support the continuity of diabetes care, particularly among the populations the study identified who had fewer telehealth uptakes, such as younger patients and those who were not taking antidiabetic medications.

With the recent announcement that Medicare Benefits Schedule (MBS) funding for telehealth became indefinitely available (as of January 2022), further investigations on improving video consultation uptake, understanding which patients or monitoring activities are most amenable to telehealth, and long-term effects of integrating in-person and remotely provided care for patients with diabetes will be critical for both consultation modalities to be used synergistically to improve patient outcomes.

## References

[1] Chandrasekaran B, Ganesan TB (2021). Sedentarism and chronic disease risk in COVID 19 lockdown — a scoping review. Scott Med J.

[2] Grabowski D, Overgaard M, Meldgaard J (2021). Disrupted self-management and adaption to new diabetes routines: a qualitative study of how people with diabetes managed their illness during the COVID-19 lockdown. Diabetology.

[3] Egan AM, Dinneen SF (2019). What is diabetes?. Medicine (Baltimore).

[4] Royal Australian College of General Practitioners, Diabetes Australia (2020). Management of type 2 diabetes: a handbook for general practice. https://www.diabetesaustralia.com.au/for-health-professionals/best-practice-guidelines/.

[5] Alromaihi D, Alamuddin N, George S (2020). Sustainable diabetes care services during COVID-19 pandemic. Diabetes Res Clin Pract.

[6] Al-Sofiani ME, Alyusuf EY, Alharthi S (2021). Rapid implementation of a diabetes telemedicine clinic during the coronavirus disease 2019 outbreak: our protocol, experience, and satisfaction reports in Saudi Arabia. J Diabetes Sci Technol.

[7] Krahmer R, Thongprayoon C, Ruanpeng D (2021). Outpatient diabetic outcome during the covid-19 pandemic: a retrospective single center analysis. J Endocr Soc.

[8] Wong VW, Wang A, Manoharan M (2021). Utilisation of telehealth for outpatient diabetes management during COVID-19 pandemic: how did the patients fare?. Intern Med J.

[9] Park S-D, Kim N-Y, Jeon J-H (2021). Impact of urgently initiated tele-prescription due to COVID-19 on glycemic control in patients with type 2 diabetes. Korean J Intern Med.

[10] Onishi Y, Yoshida Y, Takao T (2022). Diabetes management by either telemedicine or clinic visit improved glycemic control during the coronavirus disease 2019 pandemic state of emergency in Japan. J Diabetes Investig.

[11] Tourkmani AM, ALHarbi TJ, Rsheed AMB (2021). The impact of telemedicine on patients with uncontrolled type 2 diabetes mellitus during the COVID-19 pandemic in Saudi Arabia: findings and implications. J Telemed Telecare.

[12] Duckett S, Willcox S (2015). The Australian Health Care System.

[13] Hall Dykgraaf S, Desborough J, de Toca L (2021). “A decade’s worth of work in a matter of days”: the journey to telehealth for the whole population in Australia. Int J Med Inform.

[14] Royal Australian College of General Practitioners (2015). Standards for general practices (4th edition). A template for quality care and risk management in Australian general practices. https://www.racgp.org.au/running-a-practice/practice-standards/standards-4th-edition.

[15] Pearce C, McLeod A, Patrick J (2019). Coding and classifying GP data: the POLAR project. BMJ Health Care Inform.

[16] The Commonwealth Scientific and Industrial Research Organisation Shrimp. http://ontoserver.csiro.au/shrimp.

[17] Deshpande AD, Harris-Hayes M, Schootman M (2008). Epidemiology of diabetes and diabetes-related complications. Phys Ther.

[18] Kidney Health Australia (2020). Chronic kidney disease (CKD) management in primary care: guidance and clinical tips to help detect, manage and refer patients in your practice with CKD. https://kidney.org.au/uploads/resources/CKD-Management-in-Primary-Care_handbook_2020.1.pdf.

[19] World Health Organization (2021). Anatomical Therapeutic Chemical (ATC) classification. https://www.who.int/tools/atc-ddd-toolkit/atc-classification.

[20] Australian Government Department of Health (2021). COVID-19 temporary MBS telehealth services: factsheets on the use of the temporary MBS telehealth and phone consultation item numbers. http://www.mbsonline.gov.au/internet/mbsonline/publishing.nsf/Content/Factsheet-TempBB.

[21] Royal Australian College of General Practitioners (2020). Medicare Benefits Schedule fee summary: For fellows of the Royal Australian College of General Practitioners or vocationally registered general practitioners.

[22] Chen W, Qian L, Shi J (2018). Comparing performance between log-binomial and robust Poisson regression models for estimating risk ratios under model misspecification. BMC Med Res Methodol.

[23] Zhang J, Yu KF (1998). What’s the relative risk? A method of correcting the odds ratio in cohort studies of common outcomes. JAMA.

[24] Rush KL, Howlett L, Munro A, Burton L (2018). Videoconference compared to telephone in healthcare delivery: a systematic review. Int J Med Inform.

[25] Royal Australian College of General Practitioners (2021). Electronic prescribing. https://www.racgp.org.au/running-a-practice/technology/clinical-technology/electronic-prescribing.

[26] Imai C, Hardie R-A, Franco GS (2020). Harnessing the potential of electronic general practice pathology data in Australia: an examination of the quality use of pathology for type 2 diabetes patients. Int J Med Inform.

[27] Taylor A, Caffery LJ, Gesesew HA (2021). How Australian health care services adapted to telehealth during the COVID-19 pandemic: a survey of telehealth professionals. Front Public Health.

[28] Zhai Y (2021). A call for addressing barriers to telemedicine: health disparities during the COVID-19 pandemic. Psychother Psychosom.

[29] Hardie R-A, Sezgin G, Dai Z, Georgiou A (2020). The uptake of GP telehealth services during the COVID-19 pandemic. COVID-19 General Practice Snapshot.

[30] Tchero H, Kangambega P, Briatte C (2019). Clinical effectiveness of telemedicine in diabetes mellitus: a meta-analysis of 42 randomized controlled trials. Telemed J E Health.

[31] Zhai Y-K, Zhu W-J, Cai Y-L (2014). Clinical- and cost-effectiveness of telemedicine in type 2 diabetes mellitus: a systematic review and meta-analysis. Medicine (Baltimore).

